# A Randomized Controlled Trial on the Effects of Leucine-Supplement Combined with Nutritional Counseling on Body Composition in Mix Cancer Older Men

**DOI:** 10.3390/nu16020210

**Published:** 2024-01-09

**Authors:** Jéssika D. P. Soares, Jéssika M. Siqueira, Flávia dos S. B. Brito, Gustavo D. Pimentel

**Affiliations:** 1 Faculty of Nutrition, Federal University of Goias, Goiania 74605-080, Brazil; jessika_dayane@discente.ufg.br (J.D.P.S.);; 2 Institute of Nutrition, Estadual University of Rio de Janeiro, Rio de Janeiro 20550-900, Brazil; barbosaflavia@bol.com.br

**Keywords:** leucine, cancer, nutritional counselling, body composition

## Abstract

Background: Malnutrition and metabolic alterations of cancer cachexia are often associated with negative weight loss and muscle mass wasting. In this sense, protein supplementation can be a strategy to help counteract the loss and/or maintenance of mass in these patients. The aim of this study was to evaluate the effect of leucine supplementation on body composition in outpatients with gastrointestinal tract cancer. Methods: It was a randomized, blinded, controlled, parallel trial, performed in male patients with a cancer diagnosis of the gastrointestinal tract and appendix organs undergoing chemotherapy. All the patients were allocated to one of the protocol groups: L-leucine supplement or the control group, during 8 weeks of intervention. We evaluated the body composition through bioelectrical impedance analysis, the cancer cachexia classification, and the diet intake before and after the intervention protocol. The intention-to-treat approach was performed to predict the missing values for all patients who provide any observation data. Results: The patients were an average age of 65.11 ± 7.50 years old. In the body composition analysis with patients who finished all the supplementation, we observed a significant gain in body weight (61.79.9 ± 9.02 versus 64.06 ± 9.45, *p* = 0.01), ASMM (7.64 ± 1.24 versus 7.81 ± 1.20, *p* = 0.02) in the Leucine group, whereas patients in the control did not present significant variation in these parameters. There was no significant intergroup difference. While in the analysis included the patients with intention-to-treat, we found a significant increase in body weight (*p* = 0.01), BMI (*p* = 0.01), FFM (*p* = 0.03), and ASMM (*p* = 0.01) in the Leucine group. No significant intergroup differences. These results also similar among cachectic patients. Conclusion: A balanced diet enriched with free-Leucine supplementation was able to promotes gains in body weight and lean mass in older men diagnosticated with gastrointestinal and appendix organs of digestion cancer after 8 weeks. However, the fact that most men are non-cachectic or pre-cachectic is not clear if the increase in muscle mass was due to a high intake of leucine, since no difference between groups was detected. Moreover, we know that benefits on body composition are due to adequate calorie and macronutrients consumption and that balanced feeding according to nutrition Guidelines seems crucial and must be advised during the oncological treatment.

## 1. Introduction 

Gastrointestinal tract cancer is one of the most common types of cancer worldwide, affecting millions of people each year. As the world’s population ages, the incidence of this type of cancer tends to increase. In addition, the frailty associated with aging can negatively affect the treatment and recovery of patients with gastrointestinal cancer [1,2].

One of the main challenges in the treatment of cancer of the gastrointestinal tract is malnutrition. Malnutrition and metabolic alterations of cancer cachexia are often associated with an exacerbated inflammatory response, decompensated proteolysis, negative nitrogen balance, and weight loss, especially in muscle mass [3,4,5,6]. All these changes can negatively affect the quality of life of patients, functional capacity, and the effectiveness of treatment. Therefore, it is important to investigate muscle wastage in patients with gastrointestinal cancer [7,8,9].

In this sense, protein supplementation can be a strategy to help counteract the loss and/or maintenance of mass in these patients. The current recommendations of the European Society for Parenteral and Enteral Nutrition (ESPEN) suggest a protein intake of 1.0 to 1.5 g/kg/day. However, it is not very clear which amino acids should be provided to cancer patients for this purpose [10,11,12,13]. An example of these amino acids has been found to be leucine, which is an essential amino acid that stimulates muscle protein synthesis and can help prevent muscle wasting [10,14,15,16].

Therefore, cancer of the gastrointestinal tract is a significant challenge to global public health, especially as the population ages. Malnutrition and muscle wastage are common problems associated with gastrointestinal cancer and can negatively affect patients’ treatment and recovery. Our primary outcomes were to promote the gain in the weight expressed as the increase body mass index and the muscle mass. Therefore, we hypothesize that leucine supplementation will be effective in maintaining body weight, and both lean and muscle mass in patients with gastrointestinal tract cancer. Thus, the aim of this study was to evaluate the effect of leucine supplementation on body composition in outpatients with gastrointestinal tract cancer.

## 2. Materials and Methods

The trial protocol was approved by the Ethics Committees of the Federal University of Goias (n 2.977.722) and the Cancer Hospital of Goias (n 3.553.125) in September 2017, and registered at https://ensaiosclinicos.gov.br/ using the code RBR-7ycfcg. In addition, written informed consent was obtained from all patients before study inclusion. 

### 2.1. Study Design

A randomized, blinded, controlled, parallel trial was conducted in Hospital do Cancer de Goias, Goiania, Brazil. 

### 2.2. Patients, Sample Size, and Procedures

Male patients (≥60 years old) with a cancer diagnosis of the gastrointestinal tract (esophagus, stomach, colorectal), appendix organs (liver, pancreas; for all stages (I–IV), and both adjuvant and neoadjuvant chemotherapy were eligible for inclusion. Patients were considered ineligible if they (1) were men < 59 years old; (2) had mental disorders, or cognitive or walking disabilities; (3) undergoing radiotherapy or exclusive palliative treatment; (4) consumed leucine or any protein supplementation before the study; (5) in use of a pacemaker; or (6) undergoing of enteral or parenteral nutrition therapy [17]. 

The sample size was calculated by G*power (3.1 version) software^®^ (Düsseldorf, Germany). We considered an effect size of 1.08; absolute error of 0.05; test power of 0.95 and considering two groups the total simple size was in a minimum of 40 patients, being 20 per group. 

After the written informed consent, the major research enrolled patients and conducted the assessments. First, we collected data on food intake by three food recalls both to estimate caloric-protein food intake and to compose food intake baseline data. Posteriorly, the eligible patients were stratified in two blocks into the intervention groups, according to the protein intake (<1.0 g/kg/day or ≥1.0 g/kg/day, according to ESPEN) [11,13], randomized in a 1:1 ratio [18].

### 2.3. Nutritional Intervention 

All the patients were allocated to one of the protocol groups, and were administrated 7.2 g/day of L-leucine supplement (Max Titanium^®^, Matão, Brazil) or the control of 7.2 g/day of hydrolyzed collagen (Max Titanium^®^) [19], in a total of 18 capsules/day during 8 weeks of intervention. We advised the patients to consume the supplementation divided three times (at breakfast, lunch, and dinner) a day (6 capsules/time) with pure milk and to keep the habitual intake for all the following. Furthermore, the researchers advised patients on the correct storage of the supplement, to avoid damage and contamination of the product. The original packaging of the capsules was removed so that patients would not identify themselves in the group they were allocated to.

### 2.4. Nutritional Assessment 

Parameters of this section were collected at baseline (T_0_), and the end of intervention at 8 weeks (T_1_).

#### 2.4.1. Anthropometry, Body Composition, and Cancer Cachexia Classification 

Patients were weighed by a portable electronic scale (TANITA^®^, Seattle, WA, USA). Height (m) was measured by a stadiometer (Sanny^®^, São Bernardo do Campo, Brazil), and calculated the Body Mass Index (BMI) was calculated. Body composition was assessed using octopolar bioelectrical impedance analysis (SECA^®^, Hamburg, Germany) and we obtained the data of lean mass, fat mass, visceral fat, and phase angle. To estimate the Appendicular Skeletal Muscle Mass (ASMM) by Sergi’s predictive equation [20]:

$$ASMM=-3.964+\left(0.227\times \frac{height^{2}}{R}\right)+\left(0.095\times weight\right)+\left(1.384\times sex\right)+(0.064\times Xc)$$

where R = BIA resistance in ohms; height in cm; weight in kg; gender (men: 1; women: 0); and Xc = BIA reactance in ohms. Then, with the ASMM value, we categorized the patients in (1) low muscle mass if ASMM < 7.0 kg or (2) normal if ASMM ≥ 7.0 kg, according to the Sarcopenia Consensus [7]. 

To verify the cancer cachexia staging (no cachexia, pre cachexia, and cachexia) between patients, we used the criteria: (1) weight loss ≤ 5% in the last six months; (2) weight loss > 5% or IMC < 20 and weight loss > 2% or sarcopenia by decreased ASMM (<7.26 kg/m^2^) and weight loss > 2% [8].

#### 2.4.2. Dietary Intake and Adherence to Supplementation 

We applied three 24 h recalls on non-consecutive days, including weekends, and asked patients which foods and their respective measures were ingested per meal. After, we calculated the mean energy value (kcal/day), macronutrients (percentage/day), and BCAA (leucine, valine e isoleucine—g/day) adding the calorie, protein, and amino acids from supplements, which represent the final dose of leucine. To verify the adequacy of energic-protein intake, we compared their values with the ESPEN for patients with cancer, which is, 25–30 kcal/kg for calories and ≥1.0 g/k/day for protein intakes and constitutes a normocaloric and hyperproteic diet [11,13]. All the data were calculated in a program for dietary calculation (DietPro Clínico^®^, Viçosa, Brazil). The following food composition tables were selected for the calculations: (1) Brazilian Food Composition Table (TACO), developed by the University of Campinas (UNICAMP), is a well-structured table and is periodically update; and (2) North American Table (USDA), methodology developed by the United States Department of Agriculture, is composed of a significant number of foods, being frequently updated.

The adherence to supplementation was evaluated at the end of 4 weeks and the end of 8 weeks of supplementation; in both moments, the number of capsules was taken by each patient and recorded to determine the total of capsules consumed. High compliance was considered when at least 80% of the capsules were ingested. 

### 2.5. Clinical Data 

The performance status, cancer staging, and type of chemotherapy (adjuvant or neoadjuvant) were clinical data collected by medical records. The performance status was in the Karnofsky scale; however, we converted the score to Eastern Cooperative Oncology Group (ECOG) [21]. Sociodemographic data were asked by researchers’ patients during the interview. We used these data for patients’ descriptions.

### 2.6. Statistical Analyses 

The normality assumption of data was tested using the Shapiro–Wilk test. The descriptive data were presented as mean ± standard deviation, or number (proportion %). Baseline (T_0_) comparisons (Chi-square, Fisher’s exact and independent Student’s *t*-tests) were performed to assess differences between groups. 

The intention-to-treat approach was performed to predict the missing values for all patients who provide any observation data included in the analyses to be able to use all datasets [22]. Initially, we included all patients randomized into the groups to which they were assigned and who had their first assessment before the start of the protocol. Next, we applied the linear regression mathematical model to predict the values that would be achieved by those patients who discontinued the supplementation protocol. These values were replaced by missing data, which were used in statistical analyzes with the total sample. Posteriorly, we performed the sensitivity analysis to confirm the random loss of the patients. 

In addition, we conducted the repeated-measures ANOVA to verify mean differences between groups across time and interactions (the group × time). All the analyses were performed using SPSS software version 22 (IBM Corporation, Armonk, NY, USA) at a significant level of 0.05.

## 3. Results

**Figure 1:**
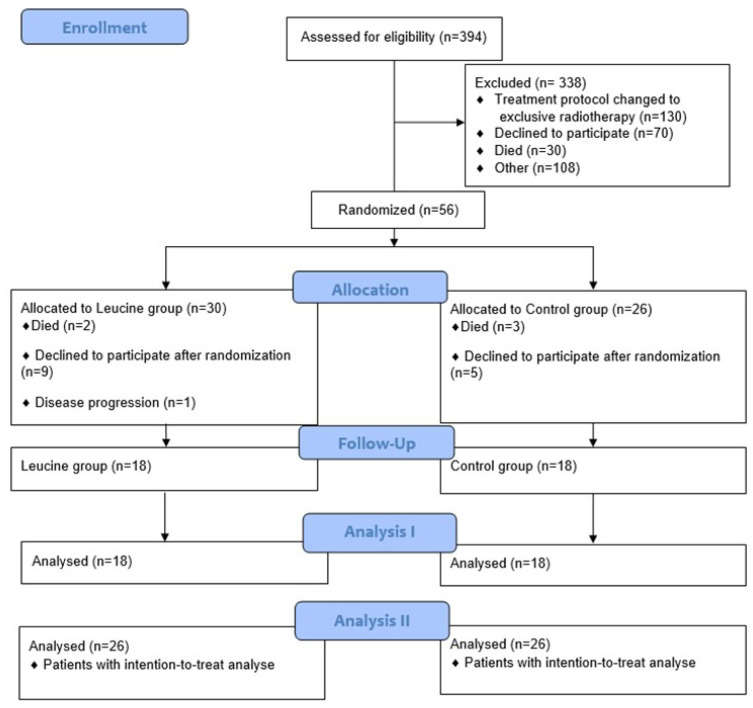
CONSORT^®^ flow diagram [18].

The study flow is presented in Figure 1. We screened 394 patients with gastrointestinal cancer and accessory organs. Of these, 338 patients met the inclusion criteria. The causes for study exclusion were undergoing radiotherapy after the screened, refusing to participate, female patients, palliative care, or starting parenteral or enteral therapy. Of the eligible 338 patients, 56 patients were randomized. 

The characteristics of baseline patients are shown in Table 1. The patients were an average age of 65.11 ± 7.50 years old, and a BMI of 22.38 ± 3.98 kg/m^2^. In the clinical data, we found a prevalence of colorectal cancer, stage disease IV, adjuvant treatment, and cachexia. No significant difference was observed between groups for any clinical and sociodemographic data. The sensibility analysis is presented in Table 1 (Supplementary Material). No significant difference was observed between groups after the insertion of intention-to-treat data.

Regarding compliance, there was no difference in adherence to supplementation (control: 96.0 ± 6.1% vs. Leucine: 98.5 ± 3.3%; *p* = 0.72). In addition, blinding was assessed to determine the efficiency of randomization. Only 40% correctly guessed their supplement in the control group, whereas only 44.5% correctly guessed their supplement in the Leucine group, with no differences between mistakes and successes among the groups (*p* = 0.87). There were no side effects found following the supplementation.

In the body composition analysis with patients who finished all the supplementation, we observed a significant gain in body weight, ASMM, and a trend to the increase in the BMI in the Leucine group, whereas patients in the control did not present significant variation in these parameters (Table 2). In addition, there was no significant intergroup difference. When the analysis included the patients with intention-to-treat, we found a significant increase in body weight, BMI, FFM, and ASMM in the Leucine group. Nonetheless, no one difference was observed in the control and intergroup interactions (Table 3). When we analyzed only the cancer cachexia patients (Table 4 and Table 5), we found similar results in body weight, BMI, FFM, and ASMM. 

The leucine intake was superior after 8 weeks, as expected. There was no difference found between different dietary intake groups or sample sizes. 

## 4. Discussion

In this present study, we observed that free-Leucine supplementation for eight weeks may promote gains in body weight and ASSM in gastrointestinal cancer and accessory organs of digestion cancer older outpatients consuming a balanced diet. Although it seems of potential clinical relevance for men with cancer, most patients are non-cachectic or pre cachectic and therefore we cannot confirm if the increase in muscle mass is due to a high intake of leucine, since no differences between groups were detected. Thus, more studies are warranted to clarify these findings. 

We evaluated the food intake and we found that caloric, protein, and carbohydrate intake are similar at baseline and remain unaltered during the intervention for both groups. However, after supplementation, the Leucine group had a higher leucine intake than the placebo group, as expected. Likewise, our previous report demonstrated that optimized adjustments of calories, carbohydrates, and total proteins were beneficial for ASMM maintenance following oncological treatment, showing that a combination of diet from macronutrients food, energy and leucine supplementation may be important to alleviating muscle wasting, since they are related to the AMP-activated protein kinase (AMPK) in the muscular tissue [10,23]. Indeed, it is known that AMPK, in the control energy balance during low calorie intake or fasting, is activated, stimulating the catabolic pathways through phosphorylation and activation of tuberous sclerosis 2 (TSC2), a negative regulator of mTORC1, which limits protein synthesis through blocking ribosomal RNA synthesis and others substrates like S6K, 4EBP-1, and EIF2, inhibiting cell circle arrest in G1 phase, and thus promoting the atrophy in muscle cells [24,25,26]. 

In fact, cancer patients display symptoms of anorexia, due to cancer cachexia, which induce the weight loss as a consequence of altered neurohormonal signals of appetite control [27]. The hypothalamus coordinates the neurons responsible to secrete anorexigenic (cocaine-and amphetamine-regulated transcript (CART) and pro-opio-melanocortin (POMC)) or orexigenic (agouti-related protein (AgRP) and neuropeptide Y (NPY)) to control food intake; however, the dysfunctional upregulating of the POMC neurons secretion results in a low food intake [9,28]. The systemic inflammation is also associated with the increase of pro-inflammatory cytokines, such as tumor-necrosis factor-α (TNF- α), interleukin- 1(IL1), interleukin-6 (IL6), and interferon-γ (IFN-γ) in the hypothalamus, promoting the inactivation of orexigenic neurons which are responsible for anorexia [9,28]^.^ Thus, the anorexia mechanism involved in its genesis, associated with pro-inflammatory cytokines, may amplify the AMPK activation and upregulate the catabolic pathway, supporting weight loss in cancer patients. 

Regarding the body composition assessment, we found an increase of ASMM and a trend in the gain of lean body mass among those patients who received a food intake enriched with leucine compared to placebo. In our study, the leucine-free supplementation appears to be effective in cancer patients consuming adequate dietary protein and regular caloric intake, as recommended by ESPEN guidelines [11,13]. Contrastingly, we found a prevalence of pre-cachexia and cachexia in both groups of supplementations; nonetheless, we did not observe any alteration in cancer cachexia after 8 weeks of intervention. 

Cancer cachexia is a common condition among cancer patients and alters the molecular process of mitochondria and energy metabolism, as cancer cells require more energy to maintain metabolism [29,30,31]. This condition promotes an increase of mitochondrial apoptosis. Likewise, muscle tissue undergoes proteolysis to release free amino acids, and to produce glucose in the Cori cycle, a metabolic waste pathway [31,32,33]. Therefore, there is a greater flow of nitrogen due to the release of alanine, an amino acid which supports the hepatic gluconeogenesis, which also contributes to muscle wastage [32].

Leucine is known for its ability to stimulate protein synthesis by mTORC1, a pathway activation in skeletal muscles, as a positive regulator of metabolism [29,34]. Recently, it was demonstrated that leucine has molecular action on mitochondrial function and structure, which may alleviate cancer-cachexia damage and improve energy production, protein synthesis, and maintenance of normal metabolism, mainly through the glycolytic pathway [29]. Cruz and colleagues (2020) used metabolomic and proteomic analyses in cachectic animal models to find information about the presence of specific metabolites and proteins in muscle tissue of tumor-bearing groups, subjected to nutritional supplementation with leucine [29]. The animals were distributed into four groups based on tumor implant of cells of Walker-256 and subjected in the diet groups: (1) (C) Control was a normoproteic diet; (2) (L) Leucine received leucine-rich diet; (3) (W) tumor-bearing received a normoproteic diet; and (4) (WL) tumor-bearing received a leucine-rich diet. After 21 days, they observed higher body and muscle weight in WL compared to the W group with no difference in tumor weight [29]. 

Concerning metabolomic and proteomic assays, Cruz and colleagues showed increased activity of mitochondria in myotubes of the L and WL groups and higher respiratory rates, improvements in the muscle ATP production in association with mitochondria, and improvements in the functional and component of the muscle cells, through an increase in the mitochondrial proteins in the muscle content [29]. In addition, leucine supplementation could modulate pro- and anti-cachectic cytokines and the proteins involved in the ubiquitin-proteasome via in-gastrocnemius muscle and could contribute to the rise in the anti-inflammatory process, in order to protect the host against the tumor effects and alleviate the cachectic status [29].

However, the randomized controlled trial with older advanced cancer patients by Storck and colleagues (2020) failed to show any increase in BMI, weight, lean mass, and muscle mass, even in a program that combined leucine supplementation with physical exercise [35]. Although they submitted their patients in a multimodal protocol as to be able to improve the nutritional status and body composition, they found an improved handgrip strength after three months in the intervention group at baseline, compared to control group (from 35.8 ± 9.8 kg to 37.6 ± 10.0 kg vs. 35.7 ± 8.8 kg to 34.0 ± 10.1 kg; *p* = 0.001) [35].

### Limtations

This study was able to assess the body composition using the bioimpedance octopolar examination, as a non-invasive tool, which is cheaper than other techniques of evaluation. In addition, we used the intent-to-treat analysis, and these findings may suggest an increased power of the test to find differences with a larger sample. However, our clinical trial had limitations: (i) we included only older men and no biochemical marker or molecular analyses to investigate inflammation levels and anabolic markers were assessed, (ii) no physical activity protocols were applied to stimulate gain of ASMM, and (iii) the lack of dosage, as well as the side effects of chemotherapy data. Additionally, our study was not thorough enough to be able to detect between-group differences.

Therefore, we propose future studies should use a similar study design to test the effects of leucine supplementation in female cancer patients on the treatments of oncological treatment.

## 5. Conclusions

A balanced diet enriched with Leucine-free supplementation may promote gains in body weight and lean mass in older men diagnosed with gastrointestinal and appendix organs of digestion cancer after 8 weeks. However, considering that most men are non-cachectic or pre-cachectic, it is not clear if the increase in muscle mass was due to a high intake of leucine, since no difference between groups was detected. Moreover, we know that the benefits on body composition are due to adequate calorie and macronutrient consumption and that balanced feeding according to nutrition Guidelines seems crucial and must be advice during the oncological treatment. Despite this, more clinical trials are necessary to establish other benefits of Leucine supplementation.

## Figures and Tables

**Table 1:** Comparison of the study patient’s characteristics between the Leucine and control groups, Goiania, Goias 2019.

Characteristics	Total (*n* = 36)	Leucine (*n* = 18)	Control (*n* = 18)	*p*
	$\bar{x}$ ± SD	$\bar{x}$ ± SD	$\bar{x}$ ± SD	
Age (years) ^§^	65.11 ± 7.50	65.22 ± 8.19	65.00 ± 7.23	0.786
BMI (kg/m^2^) ^§^	22.38 ± 3.98	22.34 ± 2.79	22.41 ± 3.64	0.933
	*n* (%)	*n* (%)	*n* (%)	
Site of primary tumor **				
Esophagus	2	1 (5.56)	1 (5.56)	0.863
Stomach	9	5 (27.78)	4 (22.22)	
Colorectal	22	10 (55.57)	12 (66.67)	
Liver	1	1 (5.56)	00 (0.00)	
Pancreas	2	1 (5.56)	1 (5.56)	
Type of chemotherapy **				
Adjuvant	35	17 (94.44)	18 (100)	0.310
Neoadjuvant	1	1 (5.56)	0 (0.00)	
Cancer stage **				
I	1	1 (5.56)	0 (0.00)	0.528
II	10	6 (33.33)	4 (22.22)	
III	11	4 (22.22)	7 (38.89)	
IV	14	7 (38.89)	7 (38.89)	
Cancer cachexia stage **				
No cachexia	7	3 (16.67)	4 (22.22)	0.210
Pre cachexia	14	7 (38.89)	7 (38.89)	
Cachexia	15	8 (44.44)	7 (38.89)	
Performance status **				
0	27	14 (77.78)	13 (72.22)	0.700
1	9	4 (22.22)	5 (27.78)	

$\bar{x}$: Mean; SD: Standard Deviation. ^§^ *p*-value obtained using Student’s *t*-test at a significance level of 5%. ** *p*-value obtained using the Chi-square test or Fisher’s exact test at a significance level of 5%.

**Table 2:** Comparison of body composition and dietary intake between the Leucine and control groups, Goiania, Goias 2019.

Variables	Treatment						*p* *
	Leucine (*n* = 18)		*p*	Control (*n* = 18)		*p*	
	Time			Time			
	T_0_	T_1_		T_0_	T_1_		
Body composition							
Weight (kg)	61.79 ± 9.02	64.06 ± 9.45	0.01	62.29 ± 10.03	62.51 ± 10.94	0.85	0.88
BMI (kg/m^2^)	22.16 ± 2.56	22.92 ± 2.62	0.09	22.44 ± 3.97	22.33 ± 4.21	0.89	0.10
FFM (kg)	48.44 ± 6.00	50.09 ± 6.49	0.11	48.93 ± 5.23	49.25 ± 6.46	0.93	0.39
FM (kg)	13.43 ± 5.64	13.96 ± 5.95	0.38	13.34 ± 6.62	13.65 ± 7.97	0.92	0.83
Visceral fat (kg)	2.68 ± 0.86	2.85 ± 0.94	0.76	2.80 ± 1.39	2.72 ± 1.46	0.98	0.48
ASMM (kg)	7.64 ± 1.24	7.81 ± 1.20	0.02	7.41 ± 1.53	7.51 ± 1.68	0.64	0.48
Dietary intake							
Energy (kcal/day)	1545.85 ± 373.66	1419.69 ± 347.34	0.23	1605.90 ± 289.86	1538.47 ± 453.91	0.25	0.68
Carbohydrate (%)	54.02 ± 7.55	53.83 ± 9.81	0.75	52.27 ± 7.54	53.52 ± 7.55	0.51	0.74
Protein (%)	20.02 ± 5.26	18.46 ± 4.98	0.14	19.82 ± 4.41	18.60 ± 4.98	0.87	0.85
Protein (g)	75.37 ± 21.32	88.12 ± 22.16	0.98	75.37 ± 21.32	80.97 ± 22.16	0.72	0.27
Protein/kg	1.22 ± 0.30	1.37 ± 0.57	0.57	1.32 ± 0.43	1.40 ± 0.40	0.42	0.78
Leucine (g/day)	4.32 ± 1.81	12.65 ± 2.00	0.01	4.78 ± 2.35	4.03 ± 2.15	0.01	0.01
Isoleucine (g/day)	2.48 ± 1.09	2.45 ± 1.20	0.30	2.83 ± 1.77	2.34 ± 1.25	0.75	0.25
Valine (g/day)	2.80 ± 1.16	2.81 ± 1.29	0.29	3.28 ± 2.23	2.63 ± 1.37	0.73	0.21

BMI: Body mass index; FFM: free fat mass; FM: fat mass; ASMM: appendicular skeletal muscle mass. * Effect of the combined interaction of treatment and time after 8 weeks of intervention.

**Table 3:** Comparison of body composition and dietary intake between the Leucine and control groups in the intention-to-treat population, Goiania, Goias 2019.

Variables	Treatment						*p* *
	Leucine (*n* = 26)		*p*	Control (*n* = 26)		*p*	
	Time			Time			
	T_0_	T_1_		T_0_	T_1_		
Body composition							
Weight (kg)	63.01 ± 1.90	65.27 ± 1.97	0.01	62.68 ± 2.02	62.51 ± 2.24	0.74	0.25
BMI (kg/m^2^)	22.31 ± 2.77	23.08 ± 2.87	0.01	22.41 ± 3.64	22.74 ± 3.93	0.89	0.23
FFM (kg)	48.56 ± 5.77	50.21 ± 6.02	0.03	49.07 ± 6.59	49.33 ± 7.08	0.90	0.20
FM (kg)	14.46 ± 7.50	14.94 ± 7.41	0.27	13.59 ± 6.66	13.84 ± 7.77	0.64	0.75
Visceral fat (kg)	2.90 ± 1.12	3.01 ± 1.08	0.88	2.69 ± 1.31	2.62 ± 1.35	0.33	0.56
ASMM (kg)	7.70 ± 1.21	7.91 ± 1.16	0.01	7.42 ± 1.46	7.62 ± 1.59	0.49	0.89
Dietary intake							
Energy (kcal/day)	1475.20 ± 422.66	1400.32 ± 302.66	0.43	1475.20 ± 332.14	1467.00 ± 406.65	0.67	0.45
Carbohydrate (%)	53.73 ± 7.12	53.54 ± 9.54	0.53	52.52 ± 8.31	54.11 ± 10.21	0.77	0.64
Protein (%)	19.91 ± 4.87	18.10 ± 5.59	0.08	18.95 ± 5.39	18.54 ± 4.19	0.56	0.53
Protein (g)	72.59 ± 23.15	87.08 ± 17.53	0.01	71.63 ± 23.15	86.91 ± 18.82	0.44	0.15
Protein/kg	1.15 ± 0.35	1.26 ± 0.53	0.01	1.18 ± 0.45	1.36 ± 0.36	0.25	0.70
Leucine (g/day)	4.21 ± 1.70	12.56 ± 1.56	0.01	4.22 ± 2.39	3.73 ± 1.93	0.19	0.01
Isoleucine (g/day)	2.37 ± 1.11	2.42 ± 1.00	0.48	2.51 ± 1.69	2.20 ± 1.09	0.88	0.24
Valine (g/day)	2.69 ± 1.22	2.75 ± 1.08	0.43	2.93 ± 2.05	2.52 ± 1.16	0.98	0.22

BMI: Body mass index; FFM: free fat mass; FM: fat mass; ASMM: appendicular skeletal muscle mass. * Effect of the combined interaction of treatment and time after 8 weeks of intervention.

**Table 4:** Comparison of body composition and diet intake between cachectic patient’s groups, Goiania, Goias 2019.

Variables	Treatment						*p* *
	Leucine (*n* = 14)		*p*	Control (*n* = 14)		*p*	
	Time			Time			
	T_0_	T_1_		T_0_	T_1_		
Body composition							
Weight (kg)	59.71 ± 8.43	62.10 ± 9.11	0.01	60.65 ± 9.97	60.97 ± 10.63	0.74	0.07
BMI (kg/m^2^)	21.52 ± 2.26	22.35 ± 2.40	0.01	21.67 ± 3.91	21.54 ± 4.00	0.79	0.06
FFM (kg)	47.18 ± 5.63	49.42 ± 6.83	0.01	47.90 ± 4.75	48.80 ± 6.26	0.50	0.28
FM (kg)	12.53 ± 5.65	12.68 ± 5.98	0.84	12.71 ± 7.27	12.16 ± 7.78	0.38	0.39
Visceral fat (kg)	2.62 ± 0.85	2.62 ± 0.87	0.63	2.53 ± 1.14	2.45 ± 1.29	0.46	0.40
ASMM (kg)	7.40 ± 1.23	7.56 ± 1.17	0.03	7.16 ± 1.49	7.21 ± 1.63	0.59	0.36
Dietary intake							
Energy (kcal/day)	1515.57 ± 406.21	1388.81 ± 365.73	0.21	1561.57 ± 310.83	1498.68 ± 456.22	0.67	0.69
Carbohydrate (%)	53.36 ± 7.07	53.56 ± 11.79	0.94	52.63 ± 8.02	54.97 ± 8.95	0.23	0.64
Protein (%)	20.49 ± 5.19	18.61 ± 5.31	0.20	19.39 ± 4.74	17.89 ± 5.30	0.16	0.82
Protein (g)	75.90 ± 23.08	93.75 ± 23.54	0.03	78.23 ± 26.28	86.56 ± 30.39	0.13	0.34
Protein/kg	1.26 ± 0.31	1.41 ± 0.42	0.12	1.31 ± 0.47	1.34 ± 0.59	0.09	0.50
Leucine (g/day)	4.34 ± 1.80	12.45 ± 2.01	0.01	4.67 ± 2.40	3.87 ± 2.35	0.15	0.01
Isoleucine (g/day)	2.46 ± 1.05	2.33 ± 1.26	0.66	2.77 ± 1.88	2.26 ± 1.37	0.20	0.53
Valine (g/day)	2.78 ± 1.15	2.66 ± 1.33	0.72	3.24 ± 2.43	2.53 ± 1.50	0.20	0.43

BMI: Body mass index; FFM: free fat mass; FM: fat mass; ASMM: appendicular skeletal muscle mass. * Effect of the combined interaction of treatment and time after 8 weeks of intervention.

**Table 5:** Comparison of body composition and dietary intake between cachectic patients’ groups in the intention-to-treat population, Goiania, Goias 2019.

Variables	Treatment						*p* *
	Leucine (*n* = 19)		*p*	Control (*n* = 19)		*p*	
	Time			Time			
	T_0_	T_1_		T_0_	T_1_		
Body composition							
Weight (kg)	61.20 ± 8.45	63.56 ± 9.00	0.01	60.72 ± 10.82	63.30 ± 11.61	0.28	0.92
BMI (kg/m^2^)	21.80 ± 2.19	22.61 ± 2.30	0.01	21.63 ± 3.72	22.26 ± 3.76	0.29	0.76
FFM (kg)	47.91 ± 5.43	49.78 ± 6.35	0.01	47.98 ± 6.46	48.72 ± 7.21	0.44	0.23
FM (kg)	13.28 ± 5.80	13.65 ± 5.96	0.52	12.71 ± 6.66	13.28 ± 7.09	0.35	0.19
Visceral fat (kg)	2.76 ± 0.85	2.78 ± 0.83	0.72	2.49 ± 1.12	2.41 ± 1.23	0.35	0.22
ASMM (kg)	7.43 ± 1.11	7.64 ± 1.06	0.01	7.15 ± 1.49	7.29 ± 1.62	0.07	0.44
Dietary intake							
Energy (kcal/day)	1475.20 ± 422.66	1400.32 ± 302.66	0.43	1475.20 ± 332.14	1467.00 ± 406.65	0.67	0.45
Carbohydrate (%)	53.50 ± 7.43	53.79 ± 10.19	0.88	52.66 ± 9.14	54.20 ± 9.49	0.32	0.61
Protein (%)	19.91 ± 4.87	18.10 ± 5.59	0.08	18.95 ± 5.39	18.54 ± 4.19	0.56	0.53
Protein (g)	75.07 ± 24.23	89.93 ± 23.38	0.04	71.82 ± 27.68	79.90 ± 30.67	0.04	0.42
Protein/kg	1.22 ± 0.35	1.40 ± 0.38	0.11	1.20 ± 0.49	1.27 ± 0.55	0.32	0.41
Leucine (g/day)	4.18 ± 1.82	12.56 ± 1.90	0.01	4.22 ± 2.50	3.65 ± 2.13	0.19	0.01
Isoleucine (g/day)	2.39 ± 1.16	2.42 ± 1.00	0.94	2.51 ± 1.69	2.20 ± 1.09	0.26	0.44
Valine (g/day)	2.72 ± 1.31	2.75 ± 1.22	0.92	2.92 ± 2.27	2.45 ± 1.31	0.28	0.40

BMI: Body mass index; FFM: free fat mass; FM: fat mass; ASMM: appendicular skeletal muscle mass. * Effect of the combined interaction of treatment and time after 8 weeks of intervention.

## Data Availability

Data are contained within the article and supplementary materials.

## Supplementary Materials

The following supporting information can be downloaded at: https://www.mdpi.com/article/10.3390/nu16020210/s1, Table S1: Sensibility analyses between the Leucine and control groups.
