# Supplementary material for: A Randomized Controlled Trial on the Effects of Leucine-Supplement Combined with Nutritional Counseling on Body Composition in Mix Cancer Older Men
Source: Nutrients. 2024 Jan 9;16(2):210. doi: 10.3390/nu16020210 (PMC10818878; doi:10.3390/nu16020210)
Supplement: Supplementary file 1 [file nutrients-16-00210-s001.zip › nutrients-2731625-supplementary.pdf]

Table S1. Sensibility analyses between the Leucine and control groups.

Characteristics	Complete (n= 36)	Incomplete (n= 20)	p
	x ± SD	x ± SD	
BMI (kg/m²) §	22.96±3.29	22.52±2.79	0.933
	n (%)	n (%)	
Site of primary tumor **			
Esophagus	2 (5.56)	2 (10)	0.215
Stomach	9 (25.00)	11(55)	
Colorectal	22 (61.1)	3 (15)	
Liver	1 (2.78)	3 (15)	
Pancreas	2(5.56)	1(5)	
Type of chemotherapy **			
Adjuvant	35 (97.2)	17(85)	0.125
Neoadjuvant	1(2.8)	3(15)	
Cancer stage **			
I	1(2.78)	0(0.0)	0.351
II	10(27.78)	2(10)	
III	11(30.56)	7(35)	
IV	14(38.88)	11(55)	
Cancer cachexia stage **			
No cachexia	7(19.44)	5(25)	0.778
Pre cachexia	14(38.89)	6(30)	
Cachexia	15(41.67)	9(45)	

x: Mean; SD: Standard Deviation

§ p-value obtained using Student's t-test at a significance level of 5%.

** p-value obtained using the Chi-square test or Fisher's exact test at a significance level of 5%.
